# The tech for the next decade: promises and challenges in genome biology

**DOI:** 10.1186/s13059-019-1695-2

**Published:** 2019-04-30

**Authors:** Iman Hajirasouliha, Hagen U. Tilgner

**Affiliations:** 1 Institute for Computational Biomedicine, Englander Institute for Precision Medicine, The Meyer Cancer Center, Department of Physiology and Biophysics, Weill Cornell Medicine of Cornell University, New York City, NY 10021 USA; 2 Center for Neurogenetics, Brain and Mind Research Institute (BMRI), Weill Cornell Medicine of Cornell University, New York City, NY 10021 USA

## Abstract

The 19th Annual Advances in Genome Biology and Technology (AGBT) meeting came back to Marco Island, Florida, and was held in the renovated venue from 27 February to 2 March 2019. The meeting showed a variety of new technology, both in wet lab and in bioinformatics. This year’s themes included single-cell technology and applications, spatially resolved gene expression measurements, new sequencing platforms, genome assembly and variation, and long and linked reads.

## Single-cell approaches and cell-type specificity: application to human disease, animal biology, and novel measurements

Aviv Regev of the Broad Institute (Cambridge, USA) classified progress in the single-cell world into measurements, inference, and perturbations. She showed (among other topics) her lab’s work in the cellular architecture of ulcerative colitis, searching for “cells of action” rather than simple genome-wide association study (GWAS) hits. Leveraging sequencing of approximately 120,000 colon cells, her lab revealed about 50 types of cells and a new cell type: the inflammatory fibroblast. Interestingly, the expression of most ulcerative colitis risk genes obtained from GWASs is also specific to particular cell types. Hadas Keren-Shaul of the Weizmann Institute (Rehovot, Israel) discussed the discovery of disease-associated microglia (DAM). In a single-cell comparison of immune cells of wild-type mice and an Alzheimer model, she found three classes of microglia, two of which do not occur in wild-type animals. These DAM overexpress a number of genes, including *TREM2*, which mediates DAM activation. Interestingly, while normal mice show only one type of microglia, humans show seven, which most likely represent different activation states. Jiannis Ragoussis of McGill University (Montreal, Canada) described a combination of single-cell copy number variation and single-cell 3′ end expression analysis. Using approximately 80,000 single cells from glioblastoma and approximately 20,000 single cells from normal fetal brain for single-cell RNA sequencing (scRNA-seq) and 30,000 glioblastoma cells for single-cell DNA sequencing (scDNA-seq), he described a progenitor population including most cycling cells of the cancer. This work suggests the possibility to define therapeutic targets for glioblastoma stem cells. Manolis Kellis from MIT (Cambridge, USA) described single-cell analyses of postmortem brains in Alzheimer’s disease. He found early cell-type-specific alterations but late global changes and large sex differences that increased with disease progression. He showed that both somatic mutations and cell type proportion changes underlie disease phenotypes, and that distinct epigenomic and transcriptional signatures underlie different sub-phenotypes of Alzheimer’s disease, pointing to much underappreciated complexity. Georg Seelig, of the University of Washington (Seattle, USA), presented his laboratory’s work on split-pool ligation-based transcriptome sequencing (SPLiT-seq). This technology uses several rounds of combinatorial barcoding to profile a large number of fixed cells or nuclei (approximately 160,000) without the need to isolate each cell, by (i) making cDNA within each cell and (ii) repeatedly barcoding the cDNAs of a few (approximately 1000) randomly chosen cells with the same barcode and repooling the cells. Application of SPLiT-seq to mouse brains at postnatal days 2 and 11 revealed more than 100 cell types. Cole Trapnell, also of the University of Washington, talked about single-cell expression profiling of whole embryos, measuring more than one million single cells simultaneously to describe mammalian organogenesis, using novel tools such as Monocle3, which is still under active development. This revealed cell populations and trajectories that appear difficult to delineate with smaller cell numbers. He furthermore described approaches to measure the impact of single-cell perturbations on hundreds of thousands of single cells and their trajectory, a technology termed “Sci-Chem”. Hagen Tilgner, one of the authors of this report, showed non-random pairing of distant exons in full-length isoforms and a method to monitor complete isoforms in thousands of single cells (single-cell isoform RNA sequencing, ScISOr-Seq). This revealed isoform expression across neuronal and glial subtypes. Jason Underwood of Pacific Biosciences (PacBio, Menlo Park, USA) presented a similar method, called single-cell ISO-Seq (ScISO-Seq), and its application to human and chimp brain organoids. He revealed polyadenylation signals that are consistent with the literature and unknown introns within 3′ UTRs that would be expected to trigger nonsense-mediated decay. Both ScISOr-Seq and ScISO-Seq use microfluidics for cDNA barcoding and long reads to deduce isoforms in single cells.

## Spatial measurements

As in AGBT 2018, spatial profiling of molecules continued to play a prominent role. In particular, Katherine McNamara of Stanford University (Stanford, USA) reported on her use of the NanoString GeoMx™ technology to profile RNA and proteins in Her2-positive breast cancer samples. She found tumor heterogeneity to increase and HER2 signaling to decrease with targeted therapy. The Broad Institute’s Sanja Vickovic reported on spatially resolved simultaneous measurements of polyadenylated host RNA and 16S bacterial RNA. She measured 11,000 host (mouse) genes and identified nine types of bacteria. In addition, she also presented a new method for in situ RNA barcoding at the subcellular 2-μm level termed high-density spatial transcriptomics (HDST). Evan Macosko, also of the Broad Institute, described the novel method of Slide-seq. Slide-seq generates cDNAs that are barcoded for their position on a slide of frozen tissue and currently achieves a resolution of 10 μm. The Macosko and Chen labs used Slide-seq to investigate mouse hippocampus and cerebellum, describing, for example, spatially restricted gene expression patterns in the Purkinje layer of the cerebellum. This spatial division was also apparent for other cerebellar cell types, including Bergmann glia and granule cells.

## Sequencing technologies

PacBio reported the new Sequel II system and on advances of circular consensus reads (CCS), which shows qualities rivaling those of Illumina. For genome assembly of the HG002 sample (Genome In A Bottle), this yields N50 s of 16.0–18.0 Mb and eases copy-number variation detection. Genapsys (Redwood City, USA) presented a soon to be released short-read sequencing platform. This product is low throughput and low cost (approximately $10,000 per machine, $300 per run) but achieves high substitution and indel accuracy, assumingly targeting the precision health market. Its low cost could potentially also be valuable in smaller laboratories for targeted variation analysis. On the other end of the spectrum (i.e., expensive and extremely high-throughput), MGI Americas (San Jose, USA) showcased the MGISEQ-T7 sequencer, a technology “created” for a world in which every individual on earth would be sequenced in the next 50 years.

## Using long-read and linked-read methods for structural variant detection

Mike Schatz of John Hopkins University (Baltimore, USA) discussed the importance of the role of structural variants (SVs) in tomato genomes in phenotypic differences. For tomatoes, a reference genome became available in 2012, but few large-scale studies followed. Schatz and colleagues identified 100 samples and sequenced them using PacBio and Nanopore long-read sequencing to study the landscape of SVs. Analysis of the first 80 long-read sequenced genomes reports about 15,000–50,000 SVs dominated by insertions and deletions. Other complex SV types are also present in their data. Arend Sidow of Stanford University described a reference-assisted assembler to detect SVs using linked reads. Short-read methods miss a large portion of genome-wide SVs. His method characterizes complex SVs missed by short reads and works well for small insertions sized less than 500 base pairs. Sidow ended his presentation with a sublime analogy: a $20,000 sedan, a $40,000 SUV, and a super luxury $400,000 car, resembling the relative cost and utility of standard Illumina short fragments, 10× genomics long fragments, and PacBio CCS, respectively, to conclude that the 40,000 SUV can get the job done.

## Genome variation

Reporting on *Drosophila* studies, Andy Clark of Cornell University (New York, USA) reported on repetitive elements and their variation among fly species. While we have come to think of repetitive sequence as long-read territory, he explained how all sequencing technologies, including Illumina, contain repeat information, which can be extracted by counting k-mers. This revealed that some repeats are equally abundant among fly species, but for others a 100-fold difference can be observed, with evidence for selective constraints on gene expression. Richard Green of University of California, Santa Cruz (UCSC, Santa Cruz, USA) showed his lab’s work on detecting archaic human ancestry algorithmically. They find that for approximately 10% of the modern human genome, one doesn’t find any trace of archaic genome influence. Approximately 1.5% of these human-specific regions have human-specific derived genetic variants. This represents regions that contribute to the differences between *Homo sapiens* and Neanderthals/Denisovans. One such region contains the splicing factor NOVA1, in which a Neanderthal valine amino acid is replaced by a *Homo sapiens* isoleucine. Organoid work suggests that this causes a splicing difference in protocadherin and other genes. Beryl Cummings of the Broad Institute leveraged the vast amount of genotype-tissue expression (GTEx) RNA-seq data in order to derive transcript-level isoform quantifications for the interpretation of putative loss-of-function variation. She finds that genetic variation in highly expressed exons is biased towards low-frequency alleles. Leveraging this expression data allowed her to filter wrongly annotated loss-of-function variation, with little impact on trustworthy pathogenic variation. Tuuli Lappalainen of the New York Genome Center (New York City, USA) reported on the use of allele-specific expression, emphasizing that allele-specific data are less affected by confounders than other approaches. She showed how the ANEVA Dosage Outlier Test (ANEVA-DOT, manuscript in preparation) captures large rare-variant effects on transcription and can be used to prioritize genes. She captures various different kinds of variants, including those introducing stop codons or frame-shifts as well as those altering splicing patterns.

## Near gap-less whole genome assembly

Mikhail Kolmogorov, a PhD student at University of California, San Diego (San Diego, USA), described Flyer, a new assembly method for error-prone long reads. He argued that the main issue in genome assembly is the characterization of all repeat families. Flyer is based on a novel repeat graph reconstruction, and similar to most long-read assemblers does not utilize a k-mer-based de Bruijn graph. He presented an algorithmic approach which results in a more accurate repeat graph. Compared with competing tools (e.g., Canu), this novel approach significantly reduces the number of misassemblies. Adam Phillippy of the National Human Genome Research Institute (Bethesda, USA) described work on a gapless human X chromosome assembly as the first human genome assembly contains hundreds of gaps in unresolved regions. To resolve this he used the, essentially haploid, CHM13 human cell line and long/linked reads, including 40× coverage with ultra-long Nanopore reads. Some reads exceeded 1 Mb and approximately 44 Gb of sequences were reads of over 100 kb. Combining their high-coverage Nanopore data with 70× coverage PacBio data, together with additional manual steps, he presented a gapless assembly for the human X chromosome and a promising whole-genome assembly (NG50 exceeding GRCh38). Karen Miga of UCSC described her effort in building high-quality reference human genomes using PromethION, the new Nanopore sequencer. They picked ten offspring from a diverse set of offspring–parent trios. Miga mentioned that the projected cost of each individual genome is about $10,000, which is lower than the cost of previous generations of long-read techniques but still one order of magnitude higher than standard short-read sequencing. She presented a pipeline for processing individual genomes from sample extraction to assembly that can be completed in one week, with the aim to produce near-gapless reference whole genomes. Edward Rice of University of Nebraska (Lincoln, USA) spoke about chromosome-length haplotigs for a hybrid cattle/yak assembly. He discussed the importance of finishing genomes and showed that 378 gaps remain in a recently improved cattle genome. His main focus was the assembly of diploid genomes using trio-binning to separate complete haplotypes. Trio-binning uses short reads from parental genomes to cluster offspring long reads into the right maternal or paternal haplotype. Rice showed that high heterozygosity eases trio-binning and presented an accurate diploid assembly of an Fl yak/cattle hybrid.

## Other applications of long-read sequencing technologies

Wilfried Haerty of the Earlham Institute (Norwich, England) reported on complex splicing patterns in the *CACNA1* genes, which are linked to a variety of diseases. *CACNA1C* has over 50 exons and spans over 700 kb. Using the MinION to investigate *CACNA1C* in multiple brain regions, his team reported 28 novel exons and increased total isoform number from about 40 to about 90. Most of the variation in *CACNA1C* occurs in the intracellular domains. Joanna Kelley of Washington State University (Pullman, USA) described temporal gene expression profiling of Grizzly bears, which are insulin resistant during hibernation but insulin sensitive at other times. Her team observed a general shutdown of metabolism-related genes during hibernation and now plans to describe isoform abundance during hibernation using PacBio.

## Beyond DNA sequence

Katherine Pollard of Gladstone Institutes (San Francisco, USA) and University of California, San Francisco (San Francisco, USA) described, among other topics, an approach to understanding protein–DNA binding based on shape rather than sequence. The algorithm is based on Gibbs sampling and finds shape-motifs de novo. She showed how DNA-binding proteins often recognize DNA shape. Interestingly, shape and sequence motifs can be located in proximity, with shape motifs providing specificity. Her lab plans to describe evolutionary conservation of shape motifs in the near future.

## Conclusion

Based on the presentations at the AGBT this year, large-scale single-cell sequencing is now widely used across different laboratories. Moreover, long-read and linked-read sequencing techniques are applied by different labs for a variety of applications such as isoform detection, de novo assembly, and structural variant characterization. We anticipate hearing about development of additional methods and large-scale studies of long-read and linked-read sequencing at next year’s meeting. Spatial profiling is becoming popular and we expect to see further improvement in technology in the near future.

## Abbreviations

CCS: Circular consensus reads
DAM: Disease-associated microglia
GWAS: Genome-wide association study
SV: Structural variant/variation

